# Effects of Two Current Great Saphenous Vein Thermal Ablation Methods on Visual Analog Scale and Quality of Life

**DOI:** 10.1155/2017/8532149

**Published:** 2017-11-16

**Authors:** Soner Sanioglu, Halit Yerebakan, Mustafa Bora Farsak

**Affiliations:** Department of Cardiovascular Surgery, Yeditepe University Hospital, Istanbul, Turkey

## Abstract

**Background:**

The aim of the study is to compare the current two endovenous thermal ablation methods by examining the effects on the visual analog scale (VAS) and the short form-36® quality of life index.

**Methods:**

Ninety-six patients who underwent unilateral endovenous thermal ablation of great saphenous vein were included. ClosureFast™ catheters were used in the RFA group and 1470 nm radial fiber laser catheters were used in the EVLA group.

**Results:**

The RFA group consisted of 41 patients and the EVLA group consisted of 55 patients. The preoperative baseline characteristics of both groups were similar. On the day of operation, VAS values were 2.8 ± 1.1 in the RFA group and 3.6 ± 1.8 in the EVLA group (*p* = 0.02). Comparisons of short form-36 parameters in both groups showed them to be similar except the pain detected at postoperative 1st week (48.1 ± 5.4 for RFA, 44.9 ± 7.6 for EVLA, *p* = 0.04).

**Conclusion:**

Results in postprocedural quality of life were found to be similar in both of the techniques. However, in terms of postoperative pain, radiofrequency ablation is still superior to the 1470 nm radial fiber laser catheters.

## 1. Introduction

According to current guidelines thermal ablation is the first choice of treatment in saphenous vein insufficiency [1, 2]. Among those, radiofrequency ablation (RFA) and endovenous laser ablation (EVLA) are the most commonly used. There is no consensus about the superiority of these techniques. Although lesser pain and complication rate was reported with RFA, using old generation laser catheters in those studies has led to question of the superiority of the RFA [3–6]. Because of this, new studies are needed with the use of new generation catheters with high wave length and new tip designs. Advances in laser catheters may alter the results and end the superiority of the RFA [7–9].

Short form-36 (SF-36®) is frequently used questionary with 36 questions in quality of life analysis. SF-36 uses 8 parameters to detect physical and mental status (physical function, physical role, pain, and general health for physical component; vitality, social role, emotional role, and mental health for mental component) [10]. Visual analog scala (VAS) is used to evaluate pain objectively. The purpose of this study is to compare the postoperative results of two current thermal ablation methods and their effects on VAS and SF-36 quality of life index.

## 2. Materials and Methods

### 2.1. Patient Groups

One hundred fifteen patients underwent great saphenous vein (GSV) thermal ablation between February 2013 and March 2016 at our institution. Among them, ninety-six patients undergoing unilateral GSV endovenous thermal ablation were enrolled in the study. Local anesthesia and mild midazolam sedation were used in all patients. Patient with previous deep vein thrombosis, accessory saphenous vein insufficiency, previous venous surgery in the same extremity, peripheral arterial disease (ABI < 0.8), patients with CEAP [11] class C4b and above, and immobilized patients were excluded from the study. The information of the patient was obtained by examining the hospital records. Patients were separated into RFA and EVLA groups.

### 2.2. Radiofrequency Ablation

The GSV was cannulated percutaneously using an 18-gauge needle with the aid of ultrasound on the knee area. A 6 F introducer sheath (INPUT Introducer Sheath, Medtronic Ireland Parkmore Business Park West, Galway, Ireland) was introduced to GSV over a guide wire, inserted through the needle used in cannulation. ClosureFast radiofrequency ablation catheter (VNUS® ClosureFast, Covidien, Dublin, Ireland) was inserted into the vein through this sheath and advanced until 2 cm below the saphenofemoral junction, guided by ultrasound. Following infiltration of perivenous field with tumescent anesthesia (500 ml saline 0.9%, 50 mg Marcaine 0.5%, 0,5 ml epinephrine 1 : 1000, and 50 mEq NaHCO_3_) ablation was performed twice for the first segment and once for the other segments with recommended energy setup.

### 2.3. Endovenous Laser Ablation

The GSV was cannulated in the same manner. A radial laser probe (Elves radial, Bolitec AG, Bonn, Germany) was inserted into the vein through sheath and advanced until 2 cm below the saphenofemoral junction, guided by ultrasound. Following infiltration of perivenous field with identical tumescent anesthesia, ablation was performed at linear endovenous energy density of 70 J/cm by a 1470 nm diode laser.

Preoperative diameter of the saphenous vein at the saphenofemoral junction, the length of ablated segment, and amount of tumescent used were recorded in both of the groups. Cutaneous varicose veins were excised in the same operating session using mini phlebectomy method. Before finishing the procedure, closure of GSV and patency of femoral vein were controlled by Doppler ultrasound. The procedure was finalized following compression bandaging of the leg.

A deep vein thrombosis prophylaxis was given using 0.1 U/kg of low molecular weight heparin to all patients and 1 gram paracetamol was ordered at the second hour postoperatively. After that compression bandages were removed and patients were dressed with grade II (23–32 mmHg) compression stockings and they were mobilized. Patients without clinical problems were discharged on the same day of surgery. Just before being discharged, their postoperative pain was evaluated by VAS (0: no pain, 10: severe pain requesting bed resting). It was advised that compression stockings be used only during daytime after the first 24 hours had passed. Compression treatment was stopped following the control examination on postoperative day 7.

### 2.4. Follow-Up Protocol

All patients were referred to the outpatient clinic at the first postoperative week, 3rd month, 6th month, and then once a year. The records of the patients were examined and the pre- and posttreatment CEAP class, venous clinical severity score [12] (VCSS), VAS, and SF-36 quality of life index values were determined. When the quality of life indexes of the patients were calculated, the Turkish version of the SF-36 form and the application at “www.sf-36.org/demos/SF-36.html” were used. Sclerotherapy was suggested to patients with CEAP class C_1_ varicose veins at the third month of follow-up. Postoperative complications and recurrences during follow-up were recorded. Control Doppler USG was not routinely performed on every patient. For this reason, recanalization rates were not evaluated in the study.

### 2.5. Statistical Analysis

Minimal clinically significant difference for postoperative VAS between groups was accepted as one (estimated standard deviation was 1,5). Cohen's *d* was 0,66 and the minimum number of patients for each groups was 38 for 80% power. Nonparametric variables of the patients were expressed as a percentage, and parametric variables were expressed as mean ± standard deviation. The normal distribution was assessed by the Kolmogorov-Smirnov test. When comparing groups, independent-sample* t*-test was used when the distribution was normal, while when it was not normal Mann–Whitney* U* test was used. The *χ*^2^ test was used to compare the nonparametric data of the groups. Fisher's exact test was preferred when the nonparametric variables were below 5 during the comparisons. For evaluating effects of the operation on the SF-36 quality of life index, when the distribution was normal the paired-sample* t*-test was used; otherwise Wilcoxon Rank Sum test was used. A value of *p* < 0.05 was considered statistically significant. All analyses were performed using the SPSS 15.0 program for Windows (SPSS Inc., Chicago, IL, USA).

## 3. Results and Discussion

### 3.1. Results

Ninety-six patients were included in the study. The RFA group consisted of 41 patients and the EVLA group consisted of 55 patients. The mean age was 46 ± 12 in the RFA group and 45 ± 10 in the EVLA group. Most of the patients in both groups consisted of female patients. At the saphenofemoral junction level, the diameter of the GSV was 8.8 ± 2.5 mm in the RFA group and 9.5 ± 2.7 mm in the EVLA group. Preoperative VCSS was 4.3 ± 1.7 in the RFA group and 4.4 ± 1.2 in the EVLA group and VAS was 5 ± 2 in the RFA group and 5.1 ± 1.8 in the EVLA group. Most of the patients in both groups were in class C_2_-C_3_. There was no significant difference between groups in terms of age, gender, comorbid disease, preoperative CEAP class, VCSS, GSV diameter, VAS, and SF-36 quality of life index (Table 1).

The success rate was 100% in both groups. The ablated segment was 27 ± 1 cm in the RFA group and 26 ± 1 cm in the EVLA group (*p* = 0.73). No statistically significant difference was found in the use of amount of tumescent anesthesia between the two groups (380 ± 22 versus 385 ± 18 ml, *p* = 0,96). The number of mini phlebectomy procedures for local varicose veins was 3.4 ± 0.7 in the RFA group and 3.6 ± 0.7 in the EVLA group (*p* = 0.3). All of the patients were discharged on the day of the procedure.

The mean follow-up was 11 ± 8 months in the RFA group and 14 ± 9 months in the EVLA group. Six months' follow-up was achieved in all the patients studied (100%), while 10 patients (20%) did not show up on their first-year control. During the follow-up period, 4 (10%) patients in the RFA group and 6 (11%) in the EVLA group had complications (*p* = 0.85). None of the patients had skin burns or deep venous thrombosis. The distribution of developing complications according to the groups is listed in Table 2. The recurrence rate was 1 (2.4%) in RFA group and 2 (3.6%) in the EVLA group (*p* = 0.73). Two of the patients were treated with high ligation and one was treated with foam sclerotherapy.

**Figure 1:**
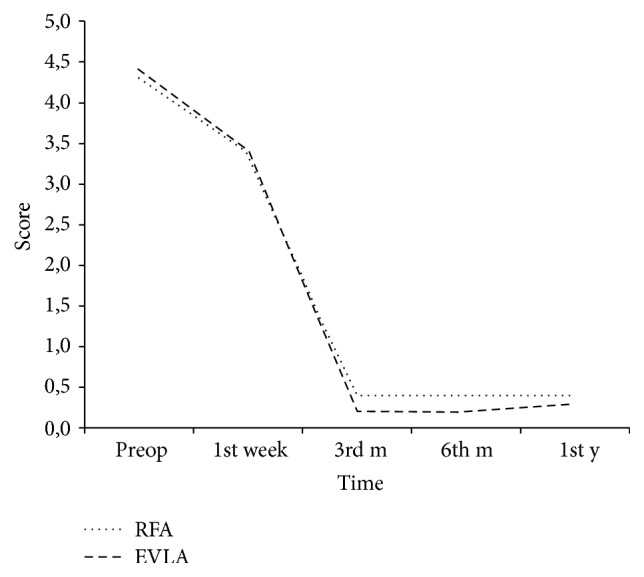
Postoperative changes for VCSS of both groups: similar improvements were detected for VCSS of both groups during follow-up (VCSS: venous clinical severity score).

The improvements in the postoperative CEAP classifications were shown in Table 3. In the first week, it was observed that the majority of the patients in both groups tended to be in the class C_0_-C_1_ and this clinical improvement was maintained throughout the follow-up period. In both groups, mean VCSS were found to decrease compared to preoperative level in the first week, but significant improvement was observed at the 3rd month (Figure 1). The delay in correction was due to mandatory compression therapy applied to all patients during the first postoperative week. When the mean VCSS obtained during follow-up were compared, no significant difference was found between the two groups.

**Figure 2:**
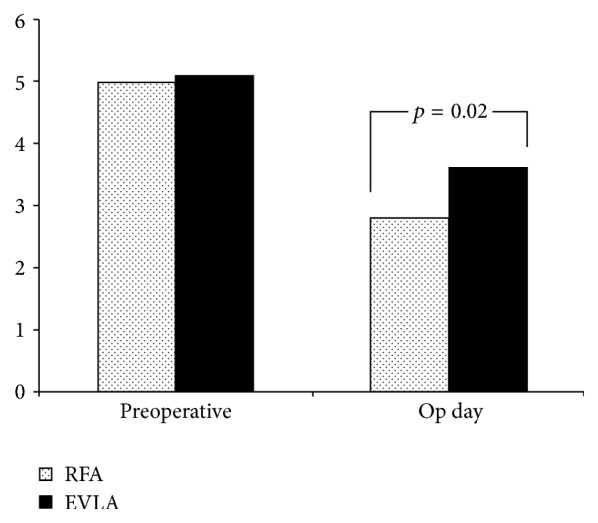
Comparison of mean preoperative and operative day VAS between two groups: preoperative VAS was similar for both groups, but operative day VAS in RFA group was significantly lower than in EVLA group (VAS: visual analog scale).

The VAS value determined on the day of operation was 2.8 ± 1.1 in the RFA group and 3.6 ± 1.8 in the EVLA group. The difference between the two groups was statistically significant (*p* = 0.02) (Figure 2).

Changes in the SF-36 quality of life index parameters following the operation are shown in Table 4. When the two groups were compared, it was seen that all parameters were similar except for the pain that was observed at 1st week (48.1 ± 5.4 for RFA and 44.9 ± 7.6 for EVLA, *p* = 0.04). When the effect of ablation on the quality of life was examined, similar changes were observed for both methods (Tables 1 and 2). In the first week, physical function, physical role, and social role parameters decreased significantly, but general health, emotional role, and mental health parameters did not change significantly in both groups. Viability increased significantly in both groups but the improvement in the pain parameters was significant in the RFA group and was not significant in the EVLA group (*p* = 0.03, *p* = 0.13). It was found that the physical component score decreased and the mental component score increased significantly in both groups. At the third month of evaluation, it was observed that the decrease in physical function, physical role, and social role disappeared in both of the groups and that the preoperative values were significantly exceeded. The general health parameters were significantly increased in both groups, while the emotional role and mental health parameters remained unchanged. It was observed that the increase in the first week observed in the vitality parameters in both groups continued increasing at the third month. Contrary to the first week, it was found that the development of the pain parameter became meaningful in both groups and the difference disappeared. It was seen that the decrease in physical component score in the first week was significantly increased at the third month and exceeded preoperative values and the increase in mental component score was decreased to below the preoperative values. At the 6th-month and 1st-year controls, it was found that the changes in the third month remained largely stable, and only the mental component score reached the preoperative values again.

### 3.2. Discussion

When the findings are examined, it can be said that both ablation methods have similar high clinical success and low complication rates. In the first week postoperatively, SF-36 resulted in a decrease in both physical component and social role scores, but, in later controls, the quality of life was found to be above preoperative levels in almost all parameters. The only significant difference between the groups was the severity of pain experienced after ablation. In the RFA group on the day of operation, the pain assessed by VAS was significantly lower than in the EVLA group (Figure 2). A significant difference was found between the two groups in the SF-36 quality of life index pain parameter on the first week and it was found that this difference between the two groups disappeared in later controls (Table 4). These results suggest that in our study group RFA caused less pain on the operation day and that this advantage continued for the first postoperative week. As is known, the level of evidence for retrospective studies is lower than that for prospective randomizations. However, the relatively homogeneous formation of the groups (Table 1), the similarity of the factors that can affect the postoperative pain, such as the ablated segment length, the amount of tumescent used, the number of phlebectomy procedures, and the anesthesia protocol increase the reliability of the results.

SF-36 is widely used in assessing quality of life. It is known that particularly physical function, physical role, pain, and general health parameters are in compliance with the severity of venous diseases, while compliance was not great in vitality, social role, emotional role, and mental health parameters that constitute the mental component [1]. Our results confirm these findings. It was observed that the improvement obtained after operation in the physical component parameters did not occur in all of the mental component parameters. Emotional role and mental health parameters were not significantly changed during treatment, and mental component, which is one of the main two components, was worsened at 3rd month, but it was found to increase again back to preoperative levels at 6th-month and 1st-year controls. It can be said that both ablation methods decrease the quality of life temporarily during the first week postoperatively, but it can be assumed that it is improved afterwards when we look at the physical component scores. In the mental component scores, the treatment modality does not seem to lead a change when we exclude the early period.

RFA revealed superior results in complication rates and postoperative pain in most of the studies [3–6]. Developments in laser catheters have led to improvements in EVLA results, and it is thought that the new catheters could end this superiority of RFA [7, 8]. However, this hypothesis has not been sufficiently questioned. There is only one study in the literature comparing RFA with current laser catheters. Bozoglan et al. used RFA and 1470 nm radial fiber laser catheter in two different extremities of the same patient and reported that EVLA was superior to RFA in terms of postoperative pain and rate of return to daily activity [9]. Our findings are not compatible with this study. In terms of postoperative pain according to our results, RFA still seems advantageous against the 1470 nm radial fiber laser catheter. Using of a rarely preferred RFA catheter was the drawback of Bozoglan's study. There could be differences in between RFA catheters as in laser catheters. The higher complication rates in the used catheters versus VNUS ClosureFast are even published in their official web site [13]. No other study comparing two RFA catheters in the literature has been found. It is clear that further studies are needed. On the other hand, it should not be overlooked that the developmental process of laser catheters is still ongoing. It has been shown that better results can be obtained with higher wave length and more different tip designs than 1470 nm radial fiber [14, 15]. While questioning which endovenous thermal ablation method is superior, this should be kept in mind.

## 4. Conclusions

As a result, two current thermal ablation methods commonly used in the treatment of GSV insufficiency have similarly high clinical success and low complication rates. Both methods provide a significant improvement in the quality of life. In terms of postoperative pain, the superiority of RFA in previous studies seems to be still maintained against 1470 nm radial fiber laser catheters. New randomized multicenter studies are needed to achieve a final result.

## Figures and Tables

**Table 1:** Comparison of preoperative variables of the groups: There is no significant difference between two groups.

	RFA (*n* = 41)	EVLA (*n* = 55)	*p* value
Age	46 ± 12	45 ± 10	0.77
Female/male	27/14	38/17	0.73
Comorbidities			
Hypertension (%)	6 (14.6)	7 (12.7)	0.78
Diabetes (%)	3 (7,3)	4 (7.3)	0.99
CAD (%)	2 (4.9)	3 (5.5)	0.90
Others (%)	3 (7.3)	5 (9.1)	0.75
CEAP class			
C_1_ (%)	1 (2)	1 (2)	0.83
C_2_ (%)	24 (59)	34 (61)	0.74
C_3_ (%)	15 (37)	19 (35)	0.83
C_4a_ (%)	1 (2)	1 (2)	0.83
GSV diameter (mm)	8.8 ± 2.5	9.5 ± 2.7	0.22
VCSS	4.3 ± 1.7	4.4 ± 1.2	0.60
Preop VAS	5 ± 2	5.1 ± 1.8	0,83
SF-36 parameters			
Physical function	48.5 ± 7.1	47.7 ± 6.6	0.39
Physical role	50.2 ± 9.8	48.6 ± 10.4	0.43
Pain	43.5 ± 9.7	43.1 ± 7.8	0.75
General health	50.3 ± 8	49.5 ± 7.0	0.27
Vitality	53.4 ± 7.5	52.8 ± 9	0.75
Social role	49.1 ± 9	49 ± 8.4	0.79
Emotional role	50.9 ± 10	50.7 ± 10.5	0.92
Mental health	46.8 ± 8	46.5 ± 9.2	0.58
* Physical component score*	*46.4 ± 8.8*	*44.8 ± 7.8*	*0.31*
* Mental component score *	*50.5 ± 9.3*	*50.6 ± 9.2*	*0.92*

CAD: coronary artery disease, VAS: visual analog scale, VCSS: venous clinical severity score, GSV: great saphenous vein, and SF-36: short form-36.

**Table 2:** Postoperative complications: There is no significant difference between two groups.

	RFA (*n* = 41)	EVLA (*n* = 55)	*p* value
Complications (%)	4 (10)	6 (11)	0.85
Hyperpigmentation (%)	1 (2.4)	2 (3.6)	
Endurance (%)	2 (4.9)	3 (5.5)	
Paresthesia (%)	1 (2.4)	—	
Cellulitis (%)	—	1 (1.8)	
Skin burn (%)	—	—	
DVT (%)	—	—	

**Table 3:** Postoperative changes of the CEAP clinical classification of the patients: both of the groups consist of mostly class C_2_-C_3_ patients. Postoperative improvements for the CEAP clinic class were maintained during follow-up.

	Preop	1st week	3rd month	6th month	1st year
	RFA (*n* = 41)				
C_0_ (%)	0 (0)	14 (34)	14 (34)	21 (52)	8 (62)
C_1_ (%)	1 (2)	26 (64)	26 (64)	18 (44)	4 (30)
C_2_ (%)	24 (59)	0 (0)	0 (0)	1 (2)	0 (0)
C_3_ (%)	15 (37)	0 (0)	0 (0)	0 (0)	0 (0)
C_4a_ (%)	1 (2)	1 (2)	1 (2)	1 (2)	1 (8)

	EVLA (*n* = 55)				
C_0_ (%)	0 (0)	25 (45)	25 (45)	32 (58)	17 (63)
C_1_ (%)	1 (2)	27 (49)	28 (51)	21 (38)	9 (33)
C_2_ (%)	34 (61)	0 (0)	1 (2)	1 (2)	0 (0)
C_3_ (%)	19 (35)	2 (4)	0 (0)	0 (0)	0 (0)
C_4a_ (%)	1 (2)	1 (2)	1 (2)	1 (2)	1 (4)

**Table 4:** Postoperative changes for SF-36 parameters: postoperative changes for the quality of life index are similar for both groups except pain parameter in the first week.

SF-36 parameters	RFA (*n* = 41)	EVLA (*n* = 55)	*p* value
1st week			
Physical function	43.6 ± 6.7	43.3 ± 6.8	0.99
Physical role	39.3 ± 7.2	38.0 ± 8.7	0.19
Pain	48.1 ± 5.4	44.9 ± 7.6	**0.04**
General health	50.5 ± 8	50.2 ± 7.5	0.58
Vitality	54 ± 7.5	53.8 ± 8.9	0.85
Social role	46 ± 8.3	44.2 ± 8.9	0.27
Emotional role	50.4 ± 10	48.6 ± 11.7	0.53
Mental health	46.8 ± 7.8	46.9 ± 8.5	0.61
*Physical component score*	*41.7 ± 5.6*	*40.4 ± 6.6*	*0.30*
*Mental component score*	*52 ± 8.3*	*51.3 ± 9.3*	*0.41*

3rd month			
Physical function	54.1 ± 5	54.3 ± 3	0.55
Physical role	55.5 ± 2.6	55.4 ± 2.2	0.58
Pain	62 ± 2.5	60.1 ± 5	0.13
General health	51.9 ± 7.7	51.1 ± 7.0	0.37
Vitality	56.2 ± 6.8	55.6 ± 7.7	0.60
Social role	51,8 ± 6.5	52.2 ± 6.9	0.50
Emotional role	51.7 ± 9	51.5 ± 9.4	0.96
Mental health	47.7 ± 7	47.5 ± 8	0.73
*Physical component score*	*56.6 ± 4.7*	*56 ± 3.6*	*0.18*
*Mental component score*	*49 ± 7.3*	*49 ± 8.2*	*0.96*

6th month			
Physical function	54.6 ± 4.6	54.6 ± 3.2	0.66
Physical role	55.7 ± 1.8	55.6 ± 2.4	0.98
Pain	61,7 ± 2.5	61.2 ± 3.7	0.71
General health	53.1 ± 7.6	52.5 ± 7.0	0.38
Vitality	56.4 ± 6.6	56.8 ± 7.8	0.95
Social role	52.1 ± 6.1	53 ± 6.3	0.34
Emotional role	53 ± 7.6	51.7 ± 8.6	0.27
Mental health	48.5 ± 6.6	48.7 ± 7.9	0.86
*Physical component score*	*56.8 ± 4.4*	*56.6 ± 3.6*	*0.32*
*Mental component score*	*50.7 ± 7*	*50 ± 8.1*	*0.96*

1st year			
Physical function	55.6 ± 2.5	55.2 ± 2.3	0.50
Physical role	56.2 ± 0.1	55.6 ± 1.3	0.85
Pain	61,1 ± 3	60.9 ± 3.6	0.96
General health	55.2 ± 8.1	53.6 ± 8.1	0.55
Vitality	56.7 ± 6.3	57.9 ± 7.6	0.53
Social role	53.8 ± 4.7	54.8 ± 5.2	0.43
Emotional role	52.9 ± 8.8	53.1 ± 6	0.79
Mental health	48.7 ± 4.3	49.5 ± 7.4	0.72
*Physical component score*	*57.8 ± 3.1*	*57 ± 3.4*	*0.39*
*Mental component score*	*50.3 ± 6.4*	*51.4 ± 7.6*	*0.72*

SF-36: short form-36.
